# The Pan-Cancer Landscape of Crosstalk Between TRP Family and Tumour Microenvironment Relevant to Prognosis and Immunotherapy Response

**DOI:** 10.3389/fimmu.2022.837665

**Published:** 2022-04-13

**Authors:** Gujie Wu, Min He, Xi Yin, Wenmaio Wang, Jiabin Zhou, Kuan Ren, Xinming Chen, Qun Xue

**Affiliations:** ^1^ Research Center of Clinical Medicine, Affiliated Hospital of Nantong University, Nantong, China; ^2^ Cardiothoracic Surgery Department, Affiliated Hospital of Nantong University, Nantong, China

**Keywords:** TRP family, pan-cancer, prognosis, tumour microenvironment, immunotherapy response

## Abstract

**Background:**

Transient Receptor Potential (TRP) channel is a kind of channel protein widely distributed in peripheral and central nervous system. They can be regulated by natural aromatic substances and serve as a therapeutic target for many diseases. However, the role and function of the TRP family genes in tumours remain unclear.

**Methods:**

Gene alterations (mutation, copy number, methylation), expression, clinical features, and prognostic value of the TRP family genes were evaluated in pan-cancer using data from The Cancer Genome Atlas and Genotype-Tissue Expression databases. TRP score was calculated by the ssGSEA function of the R package “GSVA”. The association of TRP score and the tumour microenvironment (TME), especially the tumour immune microenvironment (TIME), along with immunotherapy response were explored in-depth.

**Results:**

TRP family genes were involved in tumour progression and highly associated with poor prognosis in a variety of cancers. TRP score was positively associated with malignant pathways in pan-cancer, such as IL6–JAK–STAT3 signalling, interferon-gamma response, and inflammatory response. All pathways were closely associated with TIME. Elevated TRP score also correlated with multiple immune-related characteristics of the TIME in pan-cancer. Moreover, the TRP score was a predictive biomarker for immune checkpoint inhibitor (ICI) treatments in patients with tumours.

**Conclusions:**

TRP family genes play a key role in pan-cancer and are closely associated with TME. Patients with high TRP scores have excellent immune-activated TIME and immunotherapy sensitivity. Therefore, the TRP score could be a potential biomarker for patients with tumours treated with ICI.

## Introduction

Ion channels mediate many reactions in cell physiology and are often dysregulated in various diseases, especially in most types of tumours (1–3). Most of these channels are expressed on the cell surface and are responsible for turning intracellular signals on and off, which enable them to easily access drug targets (4). However, as an important ion channel, the involvement of transient receptor potential (TRP) family genes in tumours and their therapeutic use as targets remain unclear.

Recent research indicated the TRP channel family as potential biomarkers and/or drug targets in tumour treatment (5). For instance, TRP channels, such as TRPC1, TRPC5/6, TRPM4, TRPM7/8, TRPV1/2, TRPV4, and TRPV6 are closely associated with progression and act as novel therapeutic targets in breast invasive carcinoma (6). TRPV4 is also a proven immunomodulatory related prognostic biomarker in ovarian cancer and colorectal cancer (7, 8). In addition, TRPM8 is a potential therapeutic target in prostate adenocarcinoma (9), colon adenocarcinoma (10), breast invasive carcinoma (11), and bladder urothelial carcinoma (12).

In our study, we performed a pan-cancer systematic analysis of TRP family genes in 33 tumour types, including gene alteration (mutation, copy number, methylation), expression, clinical features, and prognostic value of the TRP family. The tumour microenvironment (TME), especially the tumour immune microenvironment (TIME), plays an important role in the development tumours (13). TIME constructed by immune cells is essential for tumour progression and cancer treatment, including chemo-, radio-, and especially immunotherapy (14–18). Thus, we calculated a TRP score and explored its association with TIME and immunotherapy response to indicate immunotherapy efficacy.

## Materials and Methods

### Data Collection

The expression profiles and clinical information of The Cancer Genome Atlas (TCGA) and Genotype-Tissue Expression (GTEx) were downloaded from the UCSC Xena (https://xenabrowser.net/datapages/) database. UCSC Xena is a cancer genomics data analysis platform that supports the visualization and analysis of a variety of omics data of cancer samples (18). The platform has built-in public data sets, including data from large cancer research projects such as TCGA and ICGC. The detailed sample sizes of tumour and normal tissues in the TCGA and GTEx databases (including tumour type, sample size of tumour tissues from TCGA, sample size of normal tissues from TCGA, and sample size of normal tissues from GTEx) are provided in **Supplementary Table 1**.

The immune cell infiltration data of the TCGA database were downloaded from the Immune Cell Abundance Identifier (ImmuCellAI) (http://bioinfo.life.hust.edu.cn/ImmuCellAI#!/) and TIMER2 databases (http://timer.cistrome.org/). The immunotherapy datasets GSE135222 and GSE91061 were downloaded from the Gene Expression Omnibus (GEO) database (https://www.ncbi.nlm.nih.gov).

### Online Analysis

The gene alterations of the TRP family, including mutation, copy number, methylation, were conducted using the Gene Set Cancer Analysis (GSCA) database (http://bioinfo.life.hust.edu.cn/GSCA/#/). GSCA is an integrated database for genomic and immunogenomic gene set cancer analysis.

### TRP Score Analysis

The single-sample gene set enrichment analysis (ssGSEA) function of R v4.1.1 package ‘GSVA’ was used to calculate the TRP score of each patient in the TCGA cohort.

### Prognostic Analysis of TRP Score

Univariate Cox regression (uniCox) analyses were performed to explore the effect of TRP score on the survival of patients, including overall survival (OS), disease-specific survival (DSS), disease-free interval (DFI), and progression-free interval (PFI) indicators, in pan-cancer using the R packages ‘survminer’ and ‘survival.’

### Gene Enrichment Analysis

To explore the biological functions of the TRP family and its role in various cancers, gene set variation analysis (GSVA) enrichment analysis was performed using the R package ‘GSVA’ to evaluate the correlation between TRP score and 50 HALLMARK pathways based on the MSigDB database (http://software.broadinstitute.org/gsea/msigdb/index.jsp).

### Tumour Microenvironment Analysis

The R package ‘ESTIMATE’ was used to calculate the stromal score, immune score, and tumour purity score of each patient in the TCGA cohort. The association between the TRP score and these scores was analysed. The TME-related pathways were obtained, and pathway scores were calculated according to a previous publication (19). We further analysed the association between TRP score and immune cell infiltration, immunomodulatory genes, MHC genes, and chemokine/chemokine receptors at the pan-cancer level. The visualization of all heatmaps in this step was conducted using the ‘ggplot2’ R package.

### Statistical Analysis

All data used in this article are presented as the mean ± standard deviation (SD). Differences between various groups were analysed and compared using the Student’s *t*-test. Statistical analysis was performed using R v4.1.1 (https://www.r-project.org/). The R packages ‘ggplot2’ and ‘ggpubr’ were used for the statistics of all histograms. Pearson correlation coefficient was used in all correlation analyses. *P* < 0.05 was considered statistically significant and indicated as follow: *P < 0.05, **P < 0.01, ***P < 0.001, and ****P < 0.0001.

## Results

### mRNA Level and Prognostic Value of TRP Family

**Figure 1:**
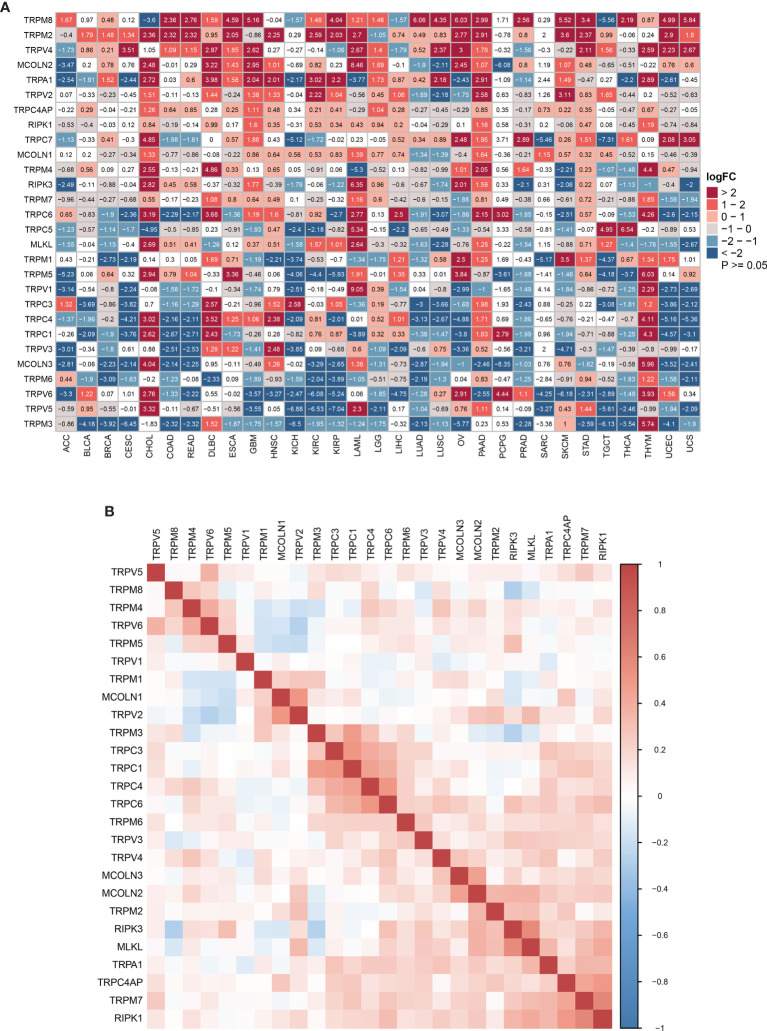
The expression of TRP family genes. **(A)** The differential expression of the TRP family in 33 tumour types based on TCGA and GTEx cohorts. The logFC and *P*-value information is presented. **(B)** The correlation between TRP family genes based on TCGA pan-cancer data. The darker the colour, the stronger the correlation.

**Figure 2:**
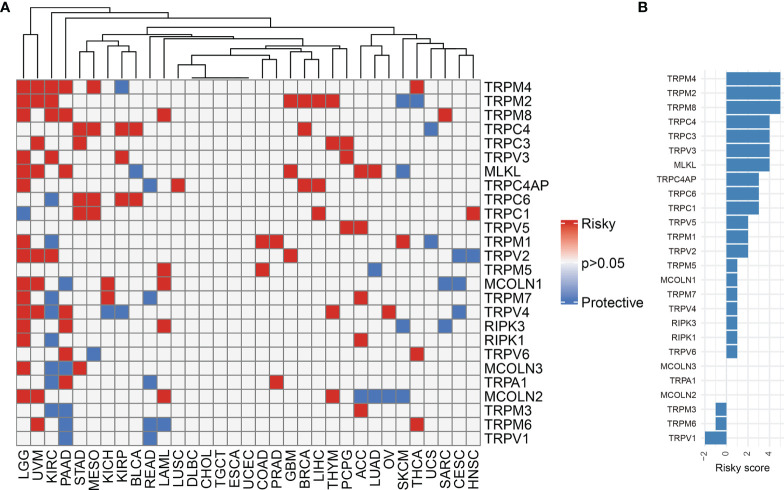
The prognostic value of TRP family genes. **(A)** Heatmap of the uniCox results of TRP family genes in each tumour type. **(B)** The risk score of each TRP family gene in pan-cancer.

We first explored the differential expression of 28 TRP family genes based on the TCGA and GTEx databases in 33 tumour types. The sample size for the cancer types is listed in **Supplementary Table 1**. As shown in **Figure 1A**, 28 TRP family genes were commonly differentially expressed in 33 tumour types. We further assessed the correlation between TRP family genes based on TCGA pan-cancer data (**Figure 1B**). Additionally, we performed a uniCox analysis of each gene in the 33 tumours (**Figure 2A**). The results indicated that most TRP family genes were risk factors in tumours. We calculated the risk scores (i.e., the number of tumours in which genes are risk factors minus the number of tumours in which genes are protective factors) and found that *TRPM4* was the most significant risk factor (**Figure 2B**). High expression of *TRPM4* predicted worse overall survival in lower-grade glioma (LGG), uveal melanoma (UVM), kidney renal clear cell carcinoma (KIRC), pancreatic adenocarcinoma (PAAD), mesothelioma (MESO), and thyroid carcinoma (THCA).

### Gene Alterations of TRP Family

**Figure 3:**
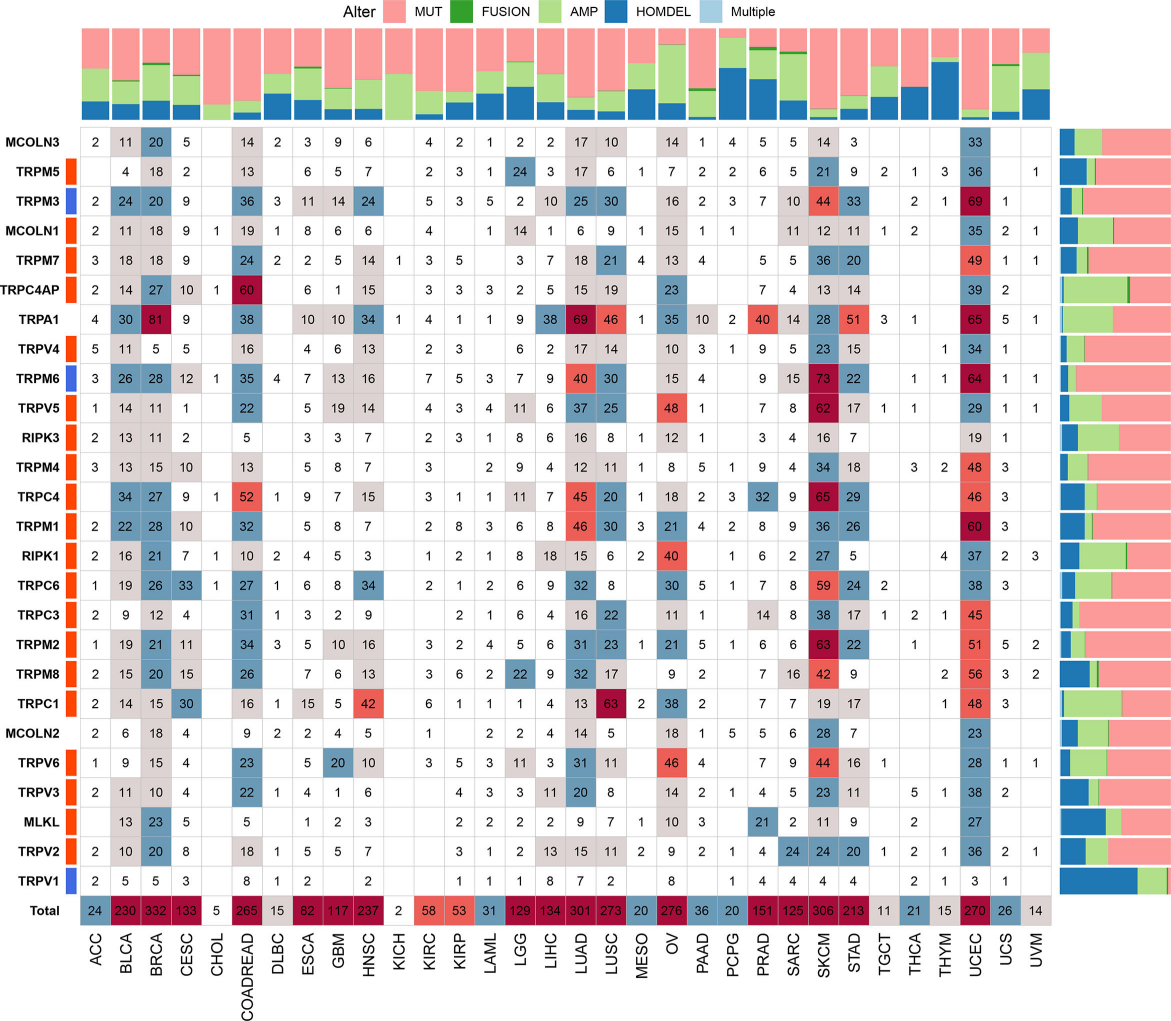
The genetic alteration numbers of TRP family genes. The genetic alteration numbers of TRP family genes in each tumour type. The number represents the occurrence of mutations, fusions, amplifications, homozygous deletions, or multiple alterations per sample.

**Figure 4:**
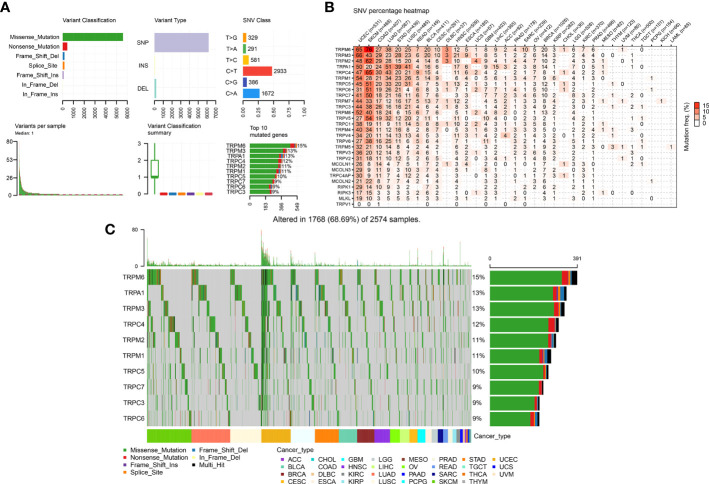
The SNV alteration of TRP family genes in pan-cancer. **(A)** The information of variant classification, variant type, SNV class, variants per sample, variant classification summary, and top 10 mutated genes in pan-cancer. **(B)** The frequency of deleterious mutations in indicated tumour types. **(C)** Oncoplot presents the mutation distribution of top 10 mutated genes in pan-cancer.

We further explored the gene alterations of the TRP family and their association with the mRNA levels. We comprehensively described the genetic alteration numbers of TRP family genes, including mutation, fusion, amplification, homozygous deletion, and multiple alterations, in the TCGA cohort (**Figure 3**). We described the variant classification, variant type, single-nucleotide variant (SNV) class, variants per sample, variant classification summary, and top10 mutated genes (**Figure 4A**). For the SNV percentage, the total deleterious mutation percentage (i.e., the number of samples with at least one deleterious mutation site/the number of samples with SNV mutation data) showed the highest deleterious mutation frequency of *TRPM6* in skin cutaneous melanoma (SKCM) (**Figure 4B**). In addition, *TRPM6* also had the highest mutation frequency (15%) among TRP family genes in pan-cancer (**Figure 4C**).

**Figure 5:**
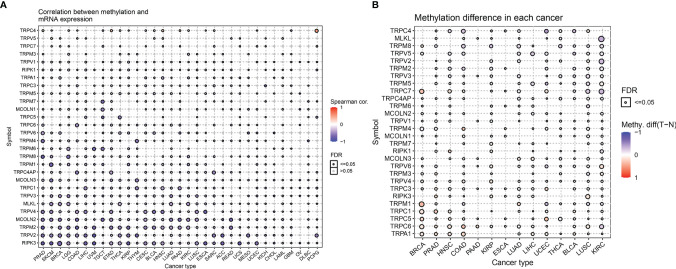
The methylation levels of TRP family genes in pan-cancer. **(A)** The correlation between methylation and mRNA level of each gene. **(B)** Summary of the methylation difference between tumour and normal samples of indicated tumour type. The fold-change and FDR are represented with the bubble colour and size, respectively. Red dots represent increased methylation in tumours and blue dots represent decreased methylation in tumours. The darker the colour of the dot, the larger the difference in methylation level.

**Figure 6:**
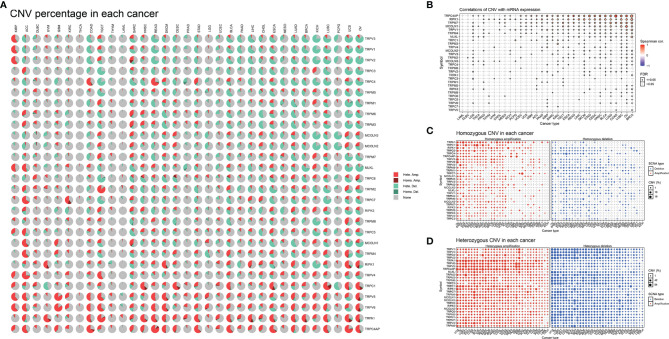
The CNV of TRP family genes in pan-cancer. **(A)** Pie plot summarising the CNV of TRP family genes in indicated tumour types. **(B)** The correlation between CNV with gene expression. **(C)** The homozygous CNV profile shows the percentage of homozygous CNV, including the percentage of homozygous amplification and deletion for each TRP family gene in each cancer. **(D)** The heterozygous CNV profile shows the percentage of heterozygous CNV, including the percentage of heterozygous amplification and deletion for each TRP family gene in each cancer.

Next, we described the methylation information of TRP family genes in pan-cancer. Results indicated that the methylation levels of TRP family genes were general negatively correlated with mRNA levels (**Figure 5A**). Subsequently, we assessed the methylation status of the TRP family genes and found that the methylation levels of TRP family genes differed between cancer types (**Figure 5B**). We further analysed the copy number variants (CNVs) of TRP family genes. The proportion of different types of CNV (including heterozygous amplification, heterozygous deletion, homozygous amplification, and homozygous deletion) of each gene in pan-cancer was shown (**Figure 6A**). The CNV of *TRPC4AP* was positively correlated with its mRNA level in 27 of 33 tumour types (**Figure 6B**). The CNV of homozygous or heterozygous amplification was positively correlated with mRNA level, and the CNV of homozygous or heterozygous deletion was negatively correlated with mRNA level (**Figures 6C, D**).

### The Estimation, Differential Distribution, and Survival Analysis of TRP Score

**Figure 7:**
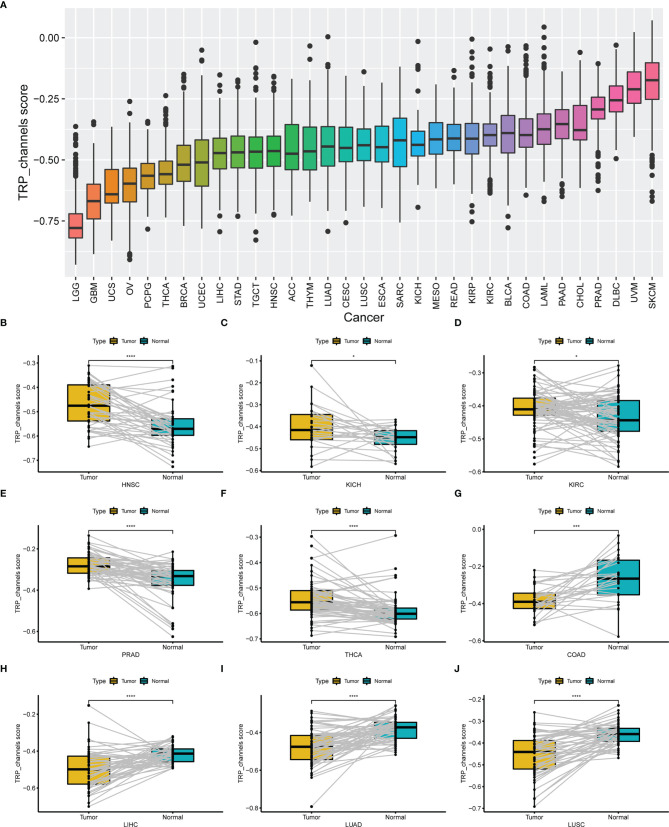
The differential distribution of TRP score. **(A)** The TRP score distribution in 33 tumour types in TCGA cohort. **(B–J)** The differential distribution of TRP score in paired tumour and adjacent normal tissues. *P < 0.05, **P < 0.01, ***P < 0.001, ****P < 0.0001.

**Figure 8:**
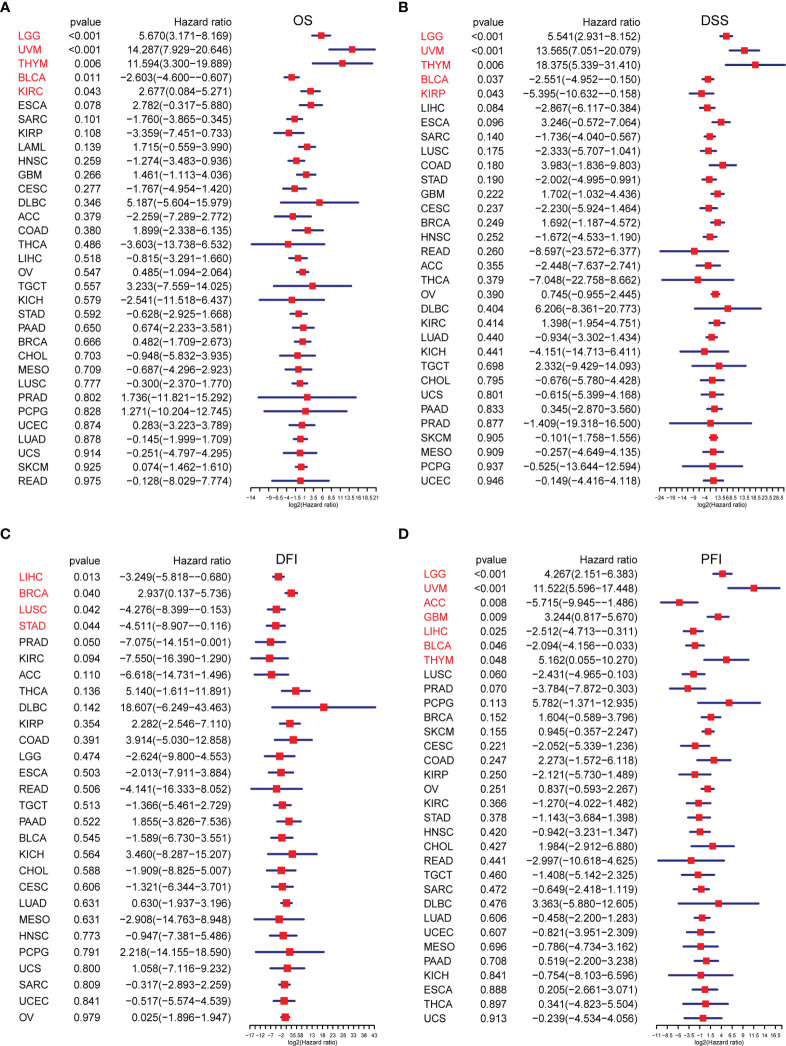
The survival analysis of TRP score. **(A–D)** Forest plots of the Cox analysis results of TRP score in pan-cancer. **(A)** Overall survival, **(B)** disease-specific survival, **(C)** disease-free interval, and **(D)** progression-free interval.

We conducted ssGSEA to estimate the TRP score across 33 tumour types in the TCGA cohort. The TRP score was the highest in SKCM and lowest in LGG (**Figure 7A**). In addition, the TRP score was higher in tumour tissues compared to the adjacent normal tissues in head and neck squamous cell carcinoma (HNSC), kidney chromophobe (KICH), KIRC, prostate adenocarcinoma (PRAD), and THCA (**Figures 7B–F**), while lower in colon adenocarcinoma (COAD), liver hepatocellular carcinoma (LIHC), lung adenocarcinoma (LUAD), and lung squamous cell carcinoma (LUSC) (**Figures 7G–J**). As for the results of uniCox analysis: (1) For OS, TRP score was a risk factor in LGG, UVM, thymoma (THYM), and KIRC, whereas a protective factor in bladder urothelial carcinoma (BLCA) (**Figure 8A**); (2) For DSS, TRP score was a risk factor in LGG, UVM, and THYM, while a protective factor in BLCA and kidney renal papillary cell carcinoma (KIRP) (**Figure 8B**); (3) For DFI, TRP score was a risk factor in breast invasive carcinoma (BRCA), whereas a protective factor in LIHC, LUSC, and stomach adenocarcinoma (STAD) (**Figure 8C**); (4) For PFI, TRP score was a risk factor in LGG, UVM, glioblastoma multiforme (GBM), and THYM, while a protective factor in adrenocortical carcinoma (ACC), LIHC, and BLCA (**Figure 8D**).

### GSVA of TRP Score

**Figure 9:**
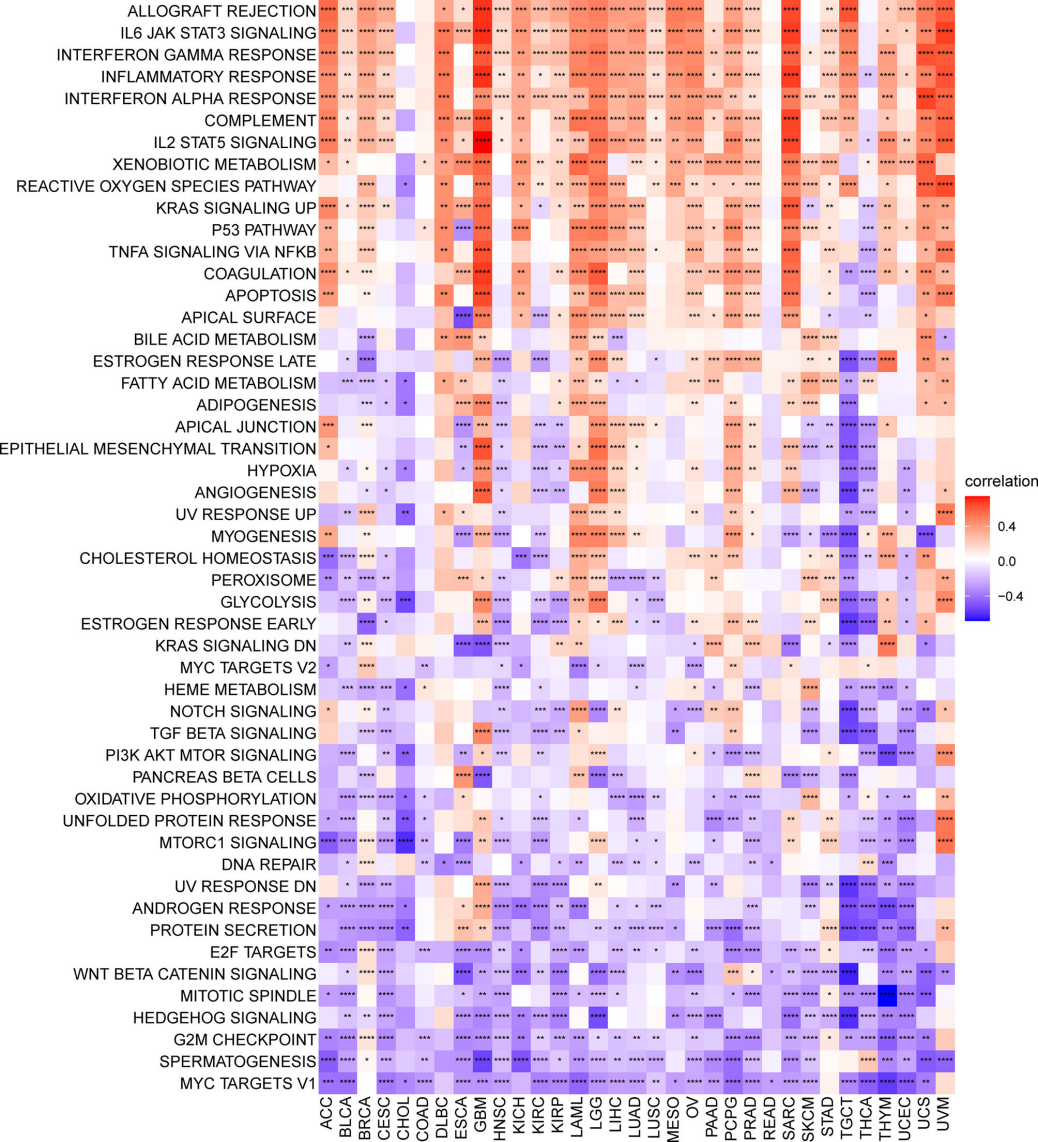
GSVA of TRP score. Heatmap of the correlation between TRP score and 50 HALLMARK pathways in pan-cancer. *P < 0.05, **P < 0.01, ***P < 0.001, ****P < 0.0001.

To analyse the potential pathways affected by the TRP score, we performed a GSVA based on 50 HALLMARK pathways. The association between the TRP score and GSVA scores in pan-cancer is shown in **Figure 9**. We observed that the TRP score was positively associated with many malignant pathways in pan-cancer, such as IL6–JAK–STAT3 signalling, interferon-gamma response, and inflammatory response. All these pathways were also closely associated with TIME.

### Relationship Between TRP Score and the TME

**Figure 10:**
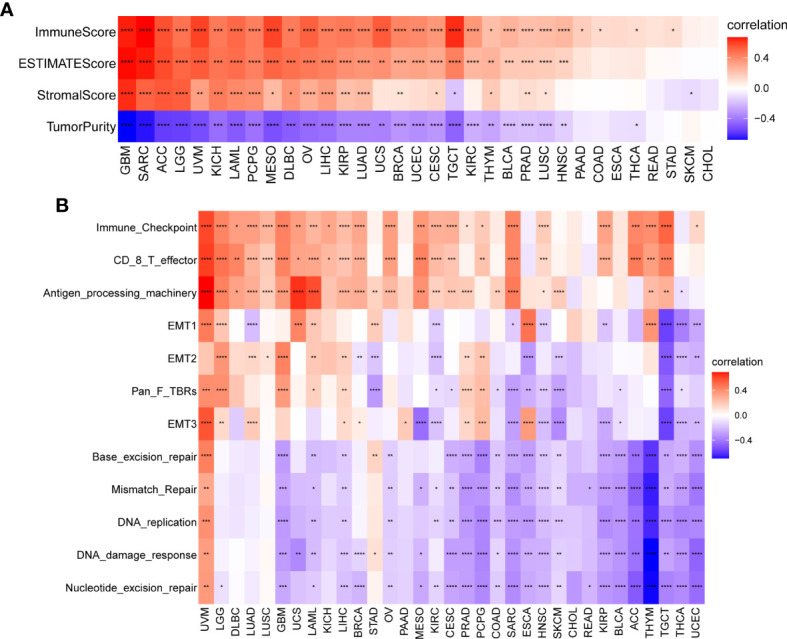
TME analysis of TRP score. **(A)** Heatmap of the correlation between TRP score and immune score, stromal score, ESTIMATE score, and tumour purity score in pan-cancer. **(B)** Heatmap of the correlation between TRP score and TME-related pathways. *P < 0.05, **P < 0.01, ***P < 0.001, ****P < 0.0001.

We further explored the association between TRP, stromal, and immune scores in pan-cancer (**Figure 10A**). Results revealed that TRP score was positively associated with an immune score, stromal score, and ESTIMATE score. We further obtained and calculated TME-related pathways according to the published paper, including immune-related pathways, stromal-related pathways, and DNA repair-related pathways. The results indicated that the TRP score was closely associated with immune-related pathways, including immune checkpoint, CD8 T effector, and antigen processing machinery, in pan-cancer (**Figure 10B**).

### Immune Infiltrating Analysis

**Figure 11:**
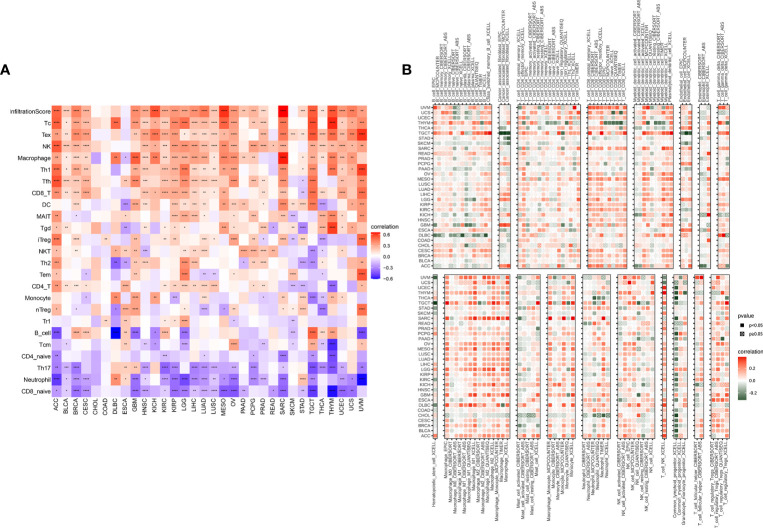
Immune infiltration analysis. **(A, B)** Correlation between TRP score and immune cell infiltration based on **(A)** ImmuCellAI or **(B)** TIMER2 database. *P < 0.05, **P < 0.01, ***P < 0.001, ****P < 0.0001.

The above results showed that the TRP score was highly correlated with the immune score. Therefore, we further explored the correlation of TRP score with immune cells in TME. Based on the ImmuCellAI database, we found that at the pan-cancer level, the TRP score is highly correlated with most immune cells, indicating an immune-activated TME (**Figure 11A**). The correlation of immune cell infiltration based on the TIMER2 database also illustrated that the TRP score had a significant positive correlation with immune-activated TME in pan-cancer (**Figure 11B**).

**Figure 12:**
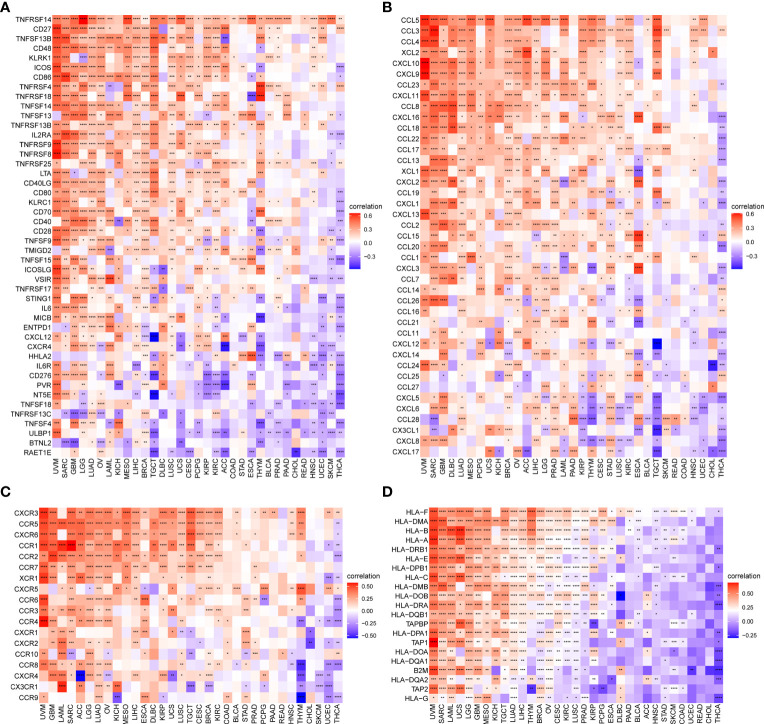
Correlation between TRP score and immune-related genes. **(A–D)**. Correlation of TRP score with **(A)** immune-activating genes, **(B)** chemokines, **(C)** chemokine receptors, or **(D)** MHC genes in pan-cancer. *P < 0.05, **P < 0.01, ***P < 0.001, ****P < 0.0001.

In addition, we observed that the TRP score was highly correlated with immune-activating genes (**Figure 12A**), chemokines (**Figure 12B**), chemokine receptors (**Figure 12C**), and MHC genes (**Figure 12D**) in most tumour types. The above results all indicated that patients with high TRP scores have elevated immune cell infiltration, which may have a positive impact on immunotherapy.

### The Association Between TRP Score and Immunotherapy Response

**Figure 13:**
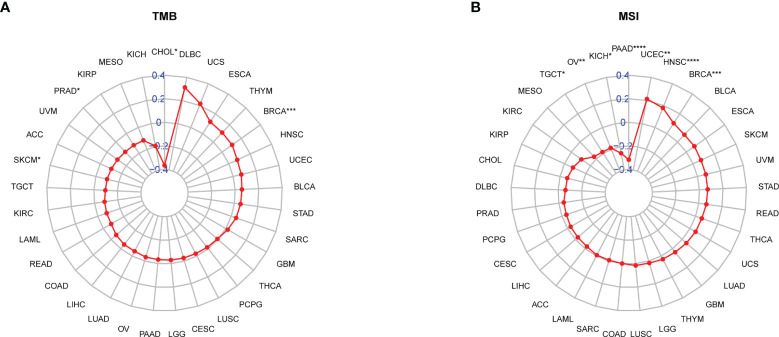
Correlation between TRP score and TMB and MSI values. **(A, B)** Radar plot of the correlation between TRP score and **(A)** TMB or **(B)** MSI. *P < 0.05, **P < 0.01, ***P < 0.001, ****P < 0.0001.

**Figure 14:**
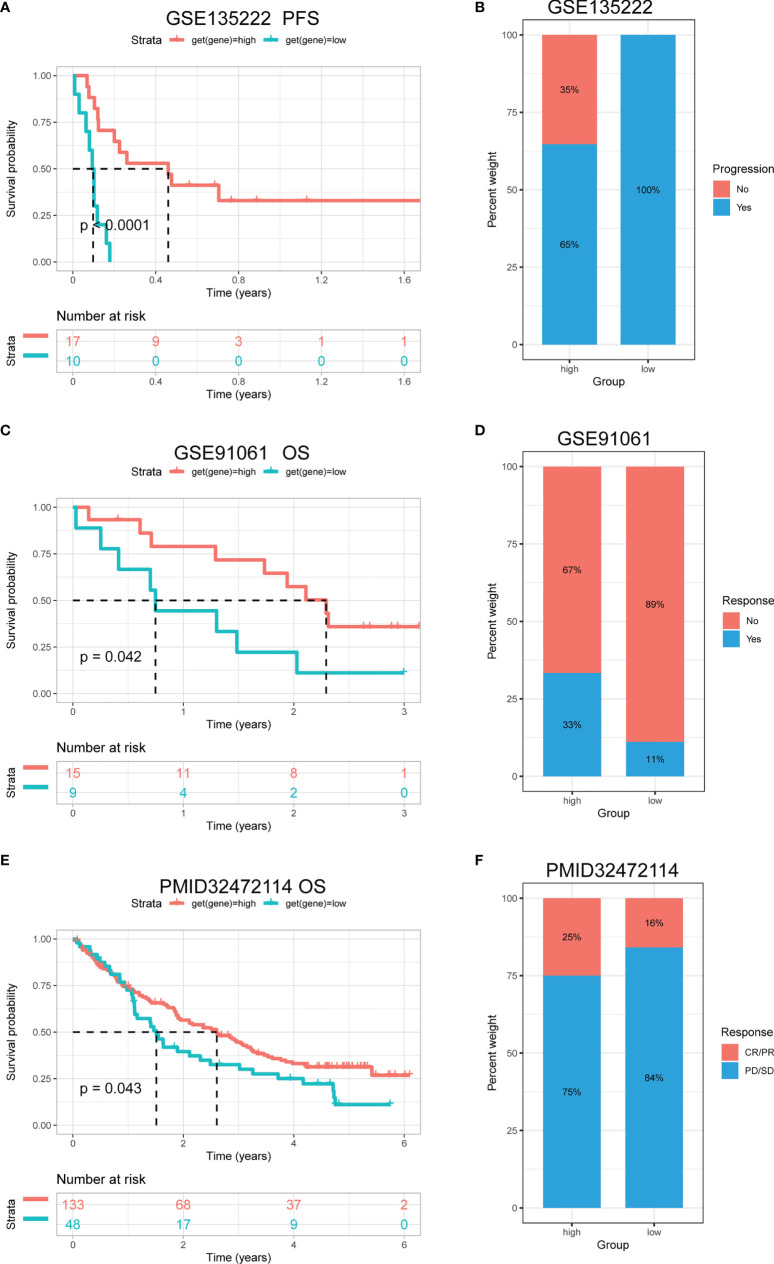
The association between TRP score and immunotherapy response. **(A)** The Kaplan-Meier PFS analysis of TRP score in GSE135222 cohort. **(B, D, F)** The percentage of responsive and progressive patients in high- and low-TRP score groups in **(B)** GSE135222, **(D)** GSE91061, and **(F)** PMID32472114 cohorts. **(C, E)** The Kaplan-Meier OS analysis of TRP score in **(C)** GSE91061 and **(E)** PMID32472114 cohorts. CR, complete response; OS, overall survival; PD, progressive disease; PFS, progression-free survival; PR, partial response; SD, stable disease.

It was reported that patients with a high tumour mutation burden (TMB) or microsatellite instability (MSI) may be sensitive to immune checkpoint inhibitor (ICI) treatment (20–22). We found that the TRP score was associated with TMB in four cancer types and MSI in seven cancer types. TRP score was positively correlated with the TMB value in BRCA (**Figure 13A**) and MSI value in BRCA, HNSC, and uterine corpus endometrial carcinoma (UCEC) (**Figure 13B**). Based on these results, we suspected that patients with high TRP scores are sensitive to immunotherapy. To verify this hypothesis, we collected immunotherapy data and calculated the TRP score. Through Kaplan-Meier analysis, we found that patients undergoing ICI treatment with high TRP scores had better OS or progression-free survival (PFS). The percentage of responsive patients was higher in the high TRP score group (**Figures 14A–F**).

## Discussion

Cancer is a multifactorial disease whose progression depends on the dysregulation of multiple pathways involved in cellular metabolism, immune evasion, avoidance of cell death, inflammation, migration, and invasion (23). Ion channels, as major regulators of cellular function, are implicated in a variety of physiological and pathophysiological relevant mechanisms (23). However, the relevance of ion channels as potential anti-tumour mechanisms is not well studied.

TRP channels are ion channels (Ca^2+^ permeable) that act as cellular sensors and are involved in the detection of temperature (heat and cold), chemical, and mechanical stimuli (24). In pathological conditions, such as cancer, dysregulation of signalling pathways can modulate the levels of certain TRP channels, thereby altering cellular sensitivity and response selection to the extracellular environment (25, 26). In this study, we explored the differential expression and prognostic value of 28 TRP family genes in 33 tumour types based on the TCGA and GTEx databases. We found that TRP family genes are extensively involved in tumour progression and serve as risk factors for most tumours— especially TRPM2, TRPM4, and TRPM8 leading to poor prognosis of patients with cancer. To further explore the potential oncogenic mechanisms of TRP family genes, we comprehensively described the number of genetic alterations in TRP family genes, including mutations, fusions, amplifications, homozygous deletions, and methylations, and found that TRPM6 through deleterious mutations and *TRPC4AP* through increased CNVs may promote tumour progression. Among these TRP family genes, it has been shown previously that TRPV2 in brain tumours (2), TRPV6 in PRAD (27), and TRPC6 in STAD (28) promote tumour progression—which validates our analysis. In addition, several reports have explored their oncogenic mechanisms. For instance, TRPM4 regulates the proliferation, migration, and invasion of prostate cancer cells by altering Ca^2+^ signalling and thus upregulates β-linked protein oncogene signalling along with its nuclear localisation (29). TRPM8-mediated Ca^2+^ influx promotes glioma progression by activating Ca^2+^ activated K^+^ ion channels with large conductance (30). TRPM2 mediates cancer cell migration *via* Ca^2+^ and Zn^2+^ (31). We also found that mutations in TRPM6 can lead to ion channel dysfunction causing primary hypomagnesaemia and secondary hypocalcaemia (32). Therefore, we hypothesize that TRP channel molecules in tumour cells promote tumour progression by regulating Ca^2+^ signalling.

Tumour development and metastasis are inextricably linked to the structure and function of the TME (33, 34). Specifically, a TME in which immune effector cells inhibit tumour growth can be used to assess the response of tumour cells to immunotherapy and has been effectively applied to influence clinical outcomes (14–18, 35, 36). We further established a TRP score based on the ssGSEA method in pan-cancer. TRP scores were higher in tumour tissue than adjacent normal tissue in HNSC, KICH, KIRC, PRAD, and THCA, while lower in COAD, LIHC, LUAD, and LUSC. In addition, high TRP scores can predict the poor prognosis of many cancers, including that of LGG, UVM, THYM, KIRC, BRCA, and GBM in accordance with previous results. Through in-depth analyses, we observed that the TRP score was positively associated with many malignant pathways in pan-cancer, such as IL6–JAK–STAT3 signalling, interferon-gamma response, and inflammatory response—all of which were closely associated with TIME. Therefore, we further explored the correlation of the TRP score with immune cells in the TME. We found that at the pan-cancer level, the TRP score is highly correlated with most immune cells, indicating an immune-activated TME. Moreover, we observed that the TRP score was highly correlated with immune-activating genes, chemokines, chemokine receptors, and MHC genes in most tumour types. The above results all indicated that patients with high TRP scores are rich in immune cell infiltration, which may have a positive impact on immunotherapy. TMB and MSI are critical biological markers of ICI response capable of predicting immunotherapy response across tumour types (20–22). Previous studies have shown that patients with high TMB/MSI had improved response rates and showed better immunotherapy treatment outcomes (37). Considering the genetic alterations of the TRP family genes in pan-cancer, we performed TMB and MSI analyses of TRP scores and collected additional immunotherapy data. Similar to the results of genetic alterations of TRP family genes in pan-cancer, our findings show that TRP scores correlate with TMB in four cancer types and MSI in seven cancer types and that patients with high TRP scores in a variety of cancers, including BRCA, are sensitive to immunotherapy. In addition, we found that patients with high TRP scores treated with ICIs had better OS or PFS in a comprehensive validation analysis of multiple immunotherapy datasets. Therefore, our results confirm that elevated TRP scores are highly correlated with pan-cancer TIME and that TRP score was a potential biomarker for ICI treatment efficacy in patients with tumours.

In conclusion, our study demonstrated that TRP family genes are risk factors for tumours and play crucial roles in tumorigenesis and progression. Elevated TRP scores were highly associated with multiple immune-related features of TIME in pan-cancer. In addition, the TRP score is a potential biomarker for treatment efficacy in patients with tumours receiving ICI therapy. The results of this study provide insight into the potential anti-tumour mechanisms of ion channels and provide a novel and effective immune anti-tumour strategy for tumour immunology research.

## Data Availability Statement

The datasets presented in this study can be found in online repositories. The names of the repository/repositories and accession number(s) can be found in the article/**Supplementary Material**.

## Author Contributions

GW conceived and designed the study. GW, MH and XY wrote the manuscript and participated in data analysis. WW and JZ participated in discussion and language editing. KR and XC reviewed the manuscript. QX provided all the funding for this study. All authors contributed to the article and approved the submitted version.

## Conflict of Interest

The authors declare that the research was conducted in the absence of any commercial or financial relationships that could be construed as a potential conflict of interest.

## Publisher’s Note

All claims expressed in this article are solely those of the authors and do not necessarily represent those of their affiliated organizations, or those of the publisher, the editors and the reviewers. Any product that may be evaluated in this article, or claim that may be made by its manufacturer, is not guaranteed or endorsed by the publisher.

## Glossary

**Table:**

ACC	adrenocortical carcinoma
BLCA	bladder urothelial carcinoma
BRCA	breast invasive carcinoma
CNVs	copy number variants
COAD	colon adenocarcinoma
DFI	disease-free interval
DSS	disease-specific survival
GBM	glioblastoma multiforme
GEO	Gene Expression Omnibus
GSCA	Gene Set Cancer Analysis
GSVA	gene set variation analysis
GTEx	Genotype-Tissue Expression
HNSC	head and neck squamous cell carcinoma
ICI	immune checkpoint inhibitor
ImmuCellAI	Immune Cell Abundance Identifier
KICH	kidney chromophobe
KIRC	kidney renal clear cell carcinoma
KIRP	kidney renal papillary cell carcinoma
LGG	lower-grade glioma
LIHC	liver hepatocellular carcinoma
LUAD	lung adenocarcinoma
LUSC	lung squamous cell carcinoma
MESO	mesothelioma
MSI	microsatellite instability
OS	overall survival
PAAD	pancreatic adenocarcinoma
PFI	progression-free interval
PFS	progression-free survival
PRAD	prostate adenocarcinoma
SD	standard deviation
SKCM	skin cutaneous melanoma
SNV	single-nucleotide variant
ssGSEA	single-sample gene set enrichment analysis
STAD	stomach adenocarcinoma
TCGA	The Cancer Genome Atlas
THCA	thyroid carcinoma
THYM	thymoma
TIME	tumour immune microenvironment
TMB	tumour mutation burden
TME	tumour microenvironment
TRP	transient receptor potential
UCEC	uterine corpus endometrial carcinoma
uniCox	Univariate Cox regression
UVM	uveal melanoma

## References

[1] Shapovalov G Ritaine A Skryma R Prevarskaya N. Role of TRP Ion Channels in Cancer and Tumorigenesis. Semin Immunopathol (2016) 38(3):357–69. doi: 10.1007/s00281-015-0525-1 26842901

[2] Chinigò G Castel H Chever O Gkika D. TRP Channels in Brain Tumors. Front Cell Dev Biol (2021) 9:617801. doi: 10.3389/fcell.2021.617801 33928077 PMC8076903

[3] Stokłosa P Borgström A Kappel S Peinelt C. TRP Channels in Digestive Tract Cancers. Int J Mol Sci (2020) 21(5). doi: 10.3390/ijms21051877 PMC7084354 32182937

[4] Koivisto A-P Belvisi MG Gaudet R Szallasi A. Advances in TRP Channel Drug Discovery: From Target Validation to Clinical Studies. Nat Rev Drug Discovery (2022) 21(1):41–59. doi: 10.1038/s41573-021-00268-4 34526696 PMC8442523

[5] Xing Y Wei X Liu Y Wang M-M Sui Z Wang X. Autophagy Inhibition Mediated by MCOLN1/TRPML1 Suppresses Cancer Metastasis *via* Regulating a ROS-Driven TP53/p53 Pathway. Autophagy (2021) 1–23. doi: 10.1080/15548627.2021.2008752. PMC9450983 34878954

[6] Saldías MP Maureira D Orellana-Serradell O Silva I Lavanderos B Cruz P. TRP Channels Interactome as a Novel Therapeutic Target in Breast Cancer. Front Oncol (2021) 11:621614. doi: 10.3389/fonc.2021.621614 34178620 PMC8222984

[7] Zhang P Xu J Zhang H Liu X-Y. Identification of TRPV4 as a Novel Target in Invasiveness of Colorectal Cancer. BMC Cancer (2021) 21(1):1264. doi: 10.1186/s12885-021-08970-7 34814869 PMC8611894

[8] Wang K Feng X Zheng L Chai Z Yu J You X. TRPV4 Is a Prognostic Biomarker That Correlates With the Immunosuppressive Microenvironment and Chemoresistance of Anti-Cancer Drugs. Front Mol Biosci (2021) 8:690500. doi: 10.3389/fmolb.2021.690500 34262942 PMC8273915

[9] Di Donato M Ostacolo C Giovannelli P Di Sarno V Gomez Monterrey IM Campiglia P. Therapeutic Potential of TRPM8 Antagonists in Prostate Cancer. Sci Rep (2021) 11(1):23232. doi: 10.1038/s41598-021-02675-4 34853378 PMC8636514

[10] Liu J-J Li L-Z Xu P. Upregulation of TRPM8 can Promote the Colon Cancer Liver Metastasis Through Mediating Akt/GSK-3 Signal Pathway. Biotechnol Appl Biochem (2022) 69(1):230–9. doi: 10.1002/bab.2102 33432591

[11] Huang Y Li S Jia Z Zhao W Zhou C Zhang R. Transient Receptor Potential Melastatin 8 (TRPM8) Channel Regulates Proliferation and Migration of Breast Cancer Cells by Activating the AMPK-ULK1 Pathway to Enhance Basal Autophagy. Front Oncol (2020) 10:573127. doi: 10.3389/fonc.2020.573127 33344232 PMC7746826

[12] Wang G Cao R Qian K Peng T Yuan L Chen L. TRPM8 Inhibition Regulates the Proliferation, Migration and ROS Metabolism of Bladder Cancer Cells. Onco Targets Ther (2020) 13:8825–35. doi: 10.2147/OTT.S257056 PMC7481304 32943886

[13] Mittal S Brown NJ Holen I. The Breast Tumor Microenvironment: Role in Cancer Development, Progression and Response to Therapy. Expert Rev Mol Diagn (2018) 18(3):227–43. doi: 10.1080/14737159.2018.1439382 29424261

[14] Deepak KGK Vempati R Nagaraju GP Dasari VR S N Rao DN. Tumor Microenvironment: Challenges and Opportunities in Targeting Metastasis of Triple Negative Breast Cancer. Pharmacol Res (2020) 153:104683. doi: 10.1016/j.phrs.2020.104683 32050092

[15] Choi J Gyamfi J Jang H Koo J. The Role of Tumor-Associated Macrophage in Breast Cancer Biology. Histol Histopathol (2018) 33(2):133–45. doi: 10.14670/HH-11-916 28681373

[16] Qiu S-Q Waaijer SJH Zwager MC de Vries EGE van der Vegt B Schröder CP. Tumor-Associated Macrophages in Breast Cancer: Innocent Bystander or Important Player? Cancer Treat Rev (2018) 70:178–89. doi: 10.1016/j.ctrv.2018.08.010 30227299

[17] Liu C Zhou X Zeng H Wu D Liu L. HILPDA Is a Prognostic Biomarker and Correlates With Macrophage Infiltration in Pan-Cancer. Front Oncol (2021) 11:597860. doi: 10.3389/fonc.2021.597860 33816230 PMC8015804

[18] Braun DA Hou Y Bakouny Z Ficial M Sant' Angelo M Forman J. Interplay of Somatic Alterations and Immune Infiltration Modulates Response to PD-1 Blockade in Advanced Clear Cell Renal Cell Carcinoma. Nat Med (2020) 26(6):909–18. doi: 10.1038/s41591-020-0839-y PMC7499153 32472114

[19] Zeng D Li M Zhou R Zhang J Sun H Shi M. Tumor Microenvironment Characterization in Gastric Cancer Identifies Prognostic and Immunotherapeutically Relevant Gene Signatures. Cancer Immunol Res (2019) 7(5):737–50. doi: 10.1158/2326-6066.CIR-18-0436 30842092

[20] Phillips D Matusiak M Gutierrez B Bhate SS Barlow GL Jiang S. Immune Cell Topography Predicts Response to PD-1 Blockade in Cutaneous T Cell Lymphoma. Nat Commun (2021) 12(1):6726. doi: 10.1038/s41467-021-26974-6 34795254 PMC8602403

[21] Scheiner B Pomej K Kirstein MM Hucke F Finkelmeier F Waidmann O. Prognosis of Patients With Hepatocellular Carcinoma Treated With Immunotherapy - Development and Validation of the CRAFITY Score. J Hepatol (2022) 76(2):353–63. doi: 10.1016/j.jhep.2021.09.035 34648895

[22] Yu G Pang Y Merchant M Kesserwan C Gangalapudi V Abdelmaksoud A. Tumor Mutation Burden, Expressed Neoantigens and the Immune Microenvironment in Diffuse Gliomas. Cancers (Basel) (2021) 13(23):6092. doi: 10.3390/cancers13236092 34885201 PMC8657099

[23] Alexander SPH Mathie A Peters JA Veale EL Striessnig J Kelly E. The Concise Guide to Pharmacology 2019/20: Ion Channels. Br J Pharmacol (2019) 176(Suppl.1):S142–228. doi: 10.1111/bph.14749 PMC6844578 31710715

[24] Berridge MJ Lipp P Bootman MD. The Versatility and Universality of Calcium Signalling. Nat Rev Mol Cell Biol (2000) 1(1):11–21. doi: 10.1038/35036035 11413485

[25] Domínguez DC Guragain M Patrauchan M. Calcium Binding Proteins and Calcium Signaling in Prokaryotes. Cell Calcium (2015) 57(3):151–65. doi: 10.1016/j.ceca.2014.12.006 25555683

[26] Clapham DE. Calcium Signaling. Cell (2007) 131(6):1047–58. doi: 10.1016/j.cell.2007.11.028 18083096

[27] Kim DY Kim SH Yang EK. *In Vitro* RNA Interference Mediated Suppression of TRPV6 Inhibits the Progression of Prostate Cancer by Modulating Cathepsin B and MMP9 Expression. Investig Clin Urol (2021) 62(4):447–54. doi: 10.4111/icu.20200511 PMC8246020 34085788

[28] Song Y Liu G Liu S Chen R Wang ±Na. Liu Z. Helicobacter Pylori Upregulates TRPC6 *via* Wnt/β-Catenin Signaling to Promote Gastric Cancer Migration and Invasion. Onco Targets Ther (2019) 12:5269–79. doi: 10.2147/OTT.S201025 PMC6613196 31308697

[29] Hong X Yu J-J. MicroRNA-150 Suppresses Epithelial-Mesenchymal Transition, Invasion, and Metastasis in Prostate Cancer Through the TRPM4-Mediated β-Catenin Signaling Pathway. Am J Physiol Cell Physiol (2019) 316(4):C463–80. doi: 10.1152/ajpcell.00142.2018 30566393

[30] Klumpp D Frank SC Klumpp L Sezgin Efe C Eckert M Edalat L. TRPM8 Is Required for Survival and Radioresistance of Glioblastoma Cells. Oncotarget (2017) 8(56):95896–913. doi: 10.18632/oncotarget.21436 PMC5707069 29221175

[31] Li F Abuarab N Sivaprasadarao A. Reciprocal Regulation of Actin Cytoskeleton Remodelling and Cell Migration by Ca2+ and Zn2+: Role of TRPM2 Channels. J Cell Sci (2016) 129(10):2016–29. doi: 10.1242/jcs.179796 27068538

[32] Wang P Qian Y Gu C Zhi X Pu L Yan D. A Functional Study for Verifying the Pathogenicity of a TRPM6 Variant of Uncertain Significance: A Novel Non-Canonical Splicing-Site Variant in Primary Hypomagnesemia With Secondary Hypocalcemia. Clin Chim Acta (2021) 523:469–75. doi: 10.1016/j.cca.2021.10.033 34755648

[33] Binnewies M Roberts EW Kersten K Chan V Fearon DF Merad M. Understanding the Tumor Immune Microenvironment (TIME) for Effective Therapy. Nat Med (2018) 24(5):541–50. doi: 10.1038/s41591-018-0014-x PMC5998822 29686425

[34] da Silva PHR Borges BC Uehara IA Soldi LR de Araújo RA Silva MJB. Chemokines and the Extracellular Matrix: Set of Targets for Tumor Development and Treatment. Cytokine (2021) 144:155548. doi: 10.1016/j.cyto.2021.155548 33972165

[35] Greten FR Grivennikov SI. Inflammation and Cancer: Triggers, Mechanisms, and Consequences. Immunity (2019) 51(1):27–41. doi: 10.1016/j.immuni.2019.06.025 31315034 PMC6831096

[36] Rundqvist H Veliça P Barbieri L Gameiro PA Bargiela D Gojkovic M. Cytotoxic T-Cells Mediate Exercise-Induced Reductions in Tumor Growth. Elife (2020) 9:e59996. doi: 10.7554/eLife.59996 33095157 PMC7584454

[37] Li R Han D Shi J Han Y Tan P Zhang R. Choosing Tumor Mutational Burden Wisely for Immunotherapy: A Hard Road to Explore. Biochim Biophys Acta Rev Cancer (2020) 1874(2):188420. doi: 10.1016/j.bbcan.2020.188420 32828886

